# Effect of different finishing/polishing protocols and aging on flexural strength, roughness, and surface characteristics of definitive restorative materials for CAD/CAM

**DOI:** 10.1007/s00784-026-07061-w

**Published:** 2026-08-11

**Authors:** Helena Nóbrega Almeida, Francisca Jennifer Duarte de Oliveira, João Vitor do Nascimento Santos, Fernanda Gurgel de Góis Moreira, Bruna Oliveira Campos, Renata Marques de Melo, Boniek Castillo Dutra Borges, Rodrigo Othávio de Assunção e Souza

**Affiliations:** 1 Department of Dentistry, Federal University of Rio Grande do Norte (UFRN), Av. Salgado Filho. 1787. Lagoa Nova, Natal, RN CEP: 59056-000 Brazil; 2 Department of Dental Materials and Prosthodontics, Institute of Science and Technology, University Estadual Paulista - UNESP, Av. Eng. Francisco Jose Longo, 777, Sao Jose dos Campos, SP 12245-000 Brazil

**Keywords:** Dental ceramics, CAD/CAM, Flexural strength, Polishing, Dental finishing

## Abstract

**Objective:**

To evaluate the effect of finishing/polishing and aging on the flexural strength, roughness, and surface characteristics of CAD/CAM restorative materials.

**Materials and methods:**

Three CAD/CAM materials were tested: Nanoceramic resin (CR), Polymer-infiltrated ceramic (PIC), and Leucite-reinforced glass-ceramic (LC). A total of 120 bars (8 × 2 × 1 mm; *n* = 15/group) were prepared and subjected to different finishing/polishing protocols (C: control; B: bur; BD: diamond bur + diamond-impregnated rubber tips; BS: diamond bur + silica-impregnated rubbers) and aging (without/with 10,000 thermocycles). Mini-flexural strength (σ) was assessed by three-point bending. Surface roughness (Sa) was analyzed using optical profilometry, while surface characterization was performed using SEM. Data were analyzed with two-way ANOVA/Tukey’s test (α = 0.05) and Weibull analysis.

**Results:**

For σ, both finishing/polishing and aging factors significantly affected PIC and LC (*p* < 0.05), whereas only finishing/polishing influenced CR. In CR, aged CR_BD had the lowest strength (112.0 ± 17.9 MPa). In PIC, aged PIC_BS (121.8 ± 21.4) showed the highest strength, while PIC_BD groups showed the lowest. In LC, the highest values were seen in aged LC_C (125.7 ± 22.9), LC_B (114.75 ± 19.09), and non-aged LC_C (108.11 ± 29.54). Sa was significantly affected by material type and finishing/polishing protocols.

**Conclusions:**

Silica-impregnated rubbers improved the flexural strength of PIC after bur finishing. The BD protocol, producing smoother surfaces, may compromise the flexural strength of LC. In contrast, CR was not significantly affected by the polishing protocol. Moreover, aging negatively impacted PIC strength, and polyurethane rubber tips impregnated with diamond particles produced smoother surfaces after bur adjustments.

**Clinical relevance:**

Within the limitations of this in vitro study, polishing protocols should be selected according to material composition, since surface smoothness does not necessarily correlate with improved mechanical performance. For CR, all polishing systems showed comparable performance; for LC, minimal intervention or no polishing better preserved mechanical performance; for PIC, silica-based polishing systems may be preferred.

## Introduction

Materials traditionally used for indirect restorations include metal, ceramics, and metal-ceramics. With advancements in computer-aided design and computer-aided manufacturing (CAD/CAM) technology, it has become possible to predictably restore teeth using a broader variety of materials, such as ceramics and composite resins [1, 2]. Ceramics stand out due to their superior aesthetics, high strength, and biocompatibility [3], presenting mechanical properties that vary according to their composition and microstructure; however, certain ceramic systems may be more susceptible to fracture and can contribute to the wear of the opposing dentition [4, 5]. Conversely, composite resins offer greater flexibility and lower abrasiveness to antagonist teeth, although they exhibit reduced wear resistance and inferior optical properties when compared to ceramics [6–9].

CAD/CAM technology has facilitated the development of novel restorative alternatives, including resin nanoceramics (CR) and polymer-infiltrated ceramic network (PIC) materials [10, 11]. CRs consist of nanomer and nanocluster fillers (20 nm silica and 4 to 11 nm zirconia), similar to conventional composite resins, which are pre-polymerized into blocks under ideal conditions of pressure and temperature during manufacturing [3]. In contrast, PIC is defined as a porous ceramic network infiltrated by a polymer phase, commonly referred to as a hybrid or resin-modified ceramic. It comprises a leucite ceramic network (86% by weight) combined with a polymer phase containing 14% by weight of urethane dimethacrylate and triethylene glycol dimethacrylate [3]. Clinical studies report excellent survival rates for both CRs (90% survival for onlays after 2 years) and PICs (97.7% after 3 years of follow-up) [12, 13]. These materials are recognized for their low abrasiveness to opposing teeth, compatibility with adhesive resin cements, high fatigue resistance, and intraoral repairability [3].

Despite the precision and advantages of CAD/CAM technology, indirect restorations frequently require surface adjustments prior to or following cementation to correct proximal and occlusal contacts or inadequate contours [14]. Nevertheless, a smooth restorative surface is paramount for the clinical longevity of the restoration [15]. Clinical adjustments inevitably increase surface roughness, compromising the aesthetic, biological, and mechanical properties of the material [14, 16]. Elevated roughness can exacerbate discoloration, promote biofilm accumulation, and increase the abrasion and wear of opposing teeth [17–19]. Furthermore, irregular or rough surfaces compromise the strength of restorative materials by predisposing them to crack propagation, chipping, and catastrophic fracture [20–22]. In addition to the immediate effects of clinical adjustments, surface degradation of these materials also occurs due to the cyclic mechanical stress of physiological mastication. To simulate these conditions, in vitro aging protocols such as water storage, thermocycling, and thermo-mechanical cycling are widely employed to replicate the oral environment [23, 24]. However, the impact of aging on the mechanical behavior, specifically the flexural strength, of these CAD/CAM materials remains unclear.

Consequently, restorations subjected to adjustments must undergo a rigorous finishing and polishing protocol, as surface quality correlates directly with clinical success [14]. Previous studies have demonstrated that these protocols dictate the surface roughness and gloss of restorations [25, 26]. Currently, there are commercially available abrasive instruments specifically designed for composite resins and others indicated for ceramics, varying in composition, shape, and grit size. Nonetheless, there is a lack of consensus regarding standard clinical or laboratory protocols to determine which instruments or sequences are most appropriate for the different types of CAD/CAM restorative materials. Moreover, despite the clinical relevance of these procedures, investigations into the effects of various finishing and polishing protocols on the flexural strength of CAD/CAM materials remain scarce, particularly concerning their interaction with material composition and aging conditions, as the majority of literature focuses predominantly on surface roughness outcomes.

Therefore, the objective of this study was to evaluate the effect of different finishing/polishing protocols and thermocycling on the flexural strength, roughness, and surface characteristics of various CAD/CAM definitive restorative materials. The null hypotheses tested were that: (a) finishing and polishing protocols would not affect the flexural strength of the CAD/CAM restorative materials; (b) aging would not influence the mechanical strength of the CAD/CAM restorative materials; and (c) finishing and polishing protocols would not alter the roughness and surface characteristics of the CAD/CAM restorative materials.

## Materials and methods

**Fig. 1:**
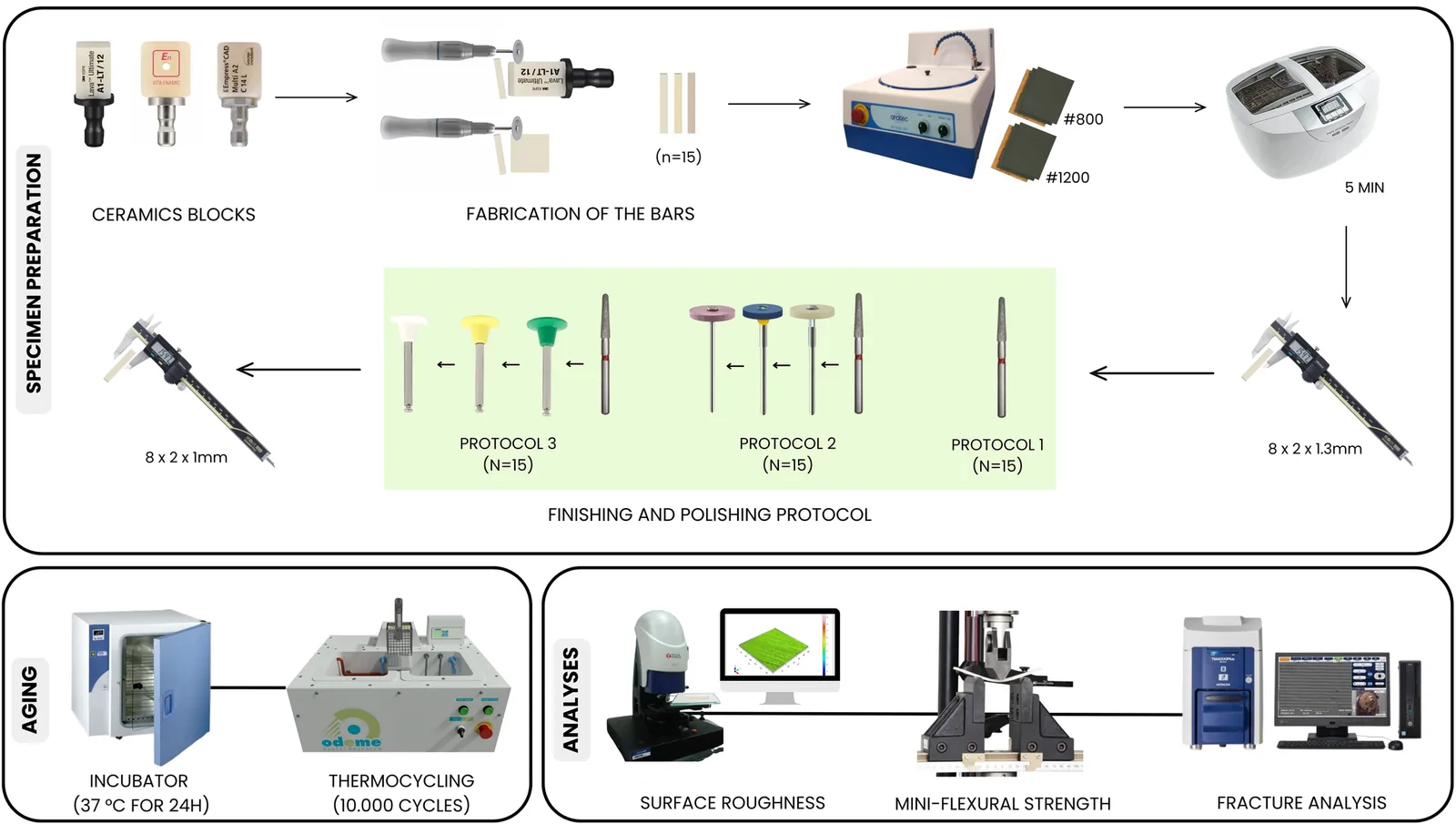
Study design flowchart

The research design is illustrated in Fig. 1, while Table 1 provides details on the materials used in this study, including their trade names, manufacturers, and compositions.

**Table 1:** List of materials, commercial brand, manufacturer, and composition

Materials	Commercial brand	Manufacturer	Composition
Polymer-Infiltrated Ceramic (PIC)	Vita Enamic	Vita Zahnfabrik, Bad Säckingen, Alemanha	86% ceramic (58–63% SiO₂, 20–23% Al₂O₃, 9–11% Na₂O, 4–6% K₂O, 0–1% ZrO₂) 14% polymer (UDMA, TEGDMA)
Nanoceramic Resin (CR)	Lava Ultimate	3 M ESPE, Saint Paul, Minnesota, USA	80% ceramic (69% SiO₂, 31% ZrO₂) 20% polymer (UDMA)
Leucite-Infiltrated Glass-Ceramic (LC)	IPS Empress CAD	Ivoclar Vivadent, Schaan, Liechtenstein	SiO_2_, Al_2_O_3_, K_2_O, Na_2_O, other oxides, pigments
Cylindrical Diamond Burs #2215	KG Sorensen	KG Sorensen, Cotia, Brasil.	Diamond Particles
Diamond-Impregnated Rubber for Ceramic Finishing and Polishing	Kit compacto premium	Dhpro, Paraná, Brasil.	Polyurethane Rubber Impregnated with Diamond Particles
Abrasive Rubber for Composite Resin Finishing and Polishing	Jiffy Polisher Disc Kit	Ultradent, São Paulo, Brasil.	Silica-Impregnated Abrasive Rubbers

### Sample size calculation

An initial sample size calculation was performed for the primary outcome (flexural strength), considering a two-tailed test, a 95% confidence level, 80% statistical power, and a 1:1 allocation ratio. The estimates were based on data from a previous study [27], which reported mean values of 434.1 ± 74.3 (control) and 352.4 ± 55.0 (experimental – burs + diamond rubber polishers), assuming an expected mean difference of 81.7. This preliminary analysis indicated a minimum of 11 specimens per group, which was increased to 15 to improve the reliability of the estimates and to enable Weibull analysis. The calculations were performed using a software program (OpenEpi Version 3.01; http://www.openepi.com).

Given the multifactorial nature of the present study, this initial estimation was considered a reference for the primary comparison of interest. Therefore, a post hoc power analysis based on the observed effect sizes obtained from the ANOVA model was additionally performed to assess the statistical power for each factor and interaction.

### Experimental design and sample preparation

For mini-flexural strength analysis, blocks (18 × 14 × 12 mm) of CAD/CAM nanoceramic resin (Lava Ultimate, 3 M ESPE, St. Paul, USA), polymer-infiltrated ceramic (Vita Enamic, Vita Zahnfabrik, Bad Säckingen, Germany), and leucite-reinforced glass-ceramic (IPS Empress CAD, Ivoclar Vivadent, Schaan, Liechtenstein) were used (*N* = 360). The samples were then randomized and allocated into 24 groups based on three factors (*n* = 15 per group): ‘Ceramic material’ (Nanoceramic resin - CR, Polymer-infiltrated ceramic - PIC, and Leucite-reinforced glass-ceramic - LC – 3 levels), ‘Finishing and polishing protocol’ (Control - C, Burrs - B, Diamond bur + ceramic rubber polisher - BD, and Diamond bur + composite resin rubber polisher – BS – 4 levels), and ‘Aging’ (with and without – 2 levels). Randomization was performed using a computer-generated randomization sequence (Research Randomizer software), by an independent researcher not involved in specimen preparation or testing, to ensure allocation concealment and minimize selection bias. A pilot study was conducted to determine the expected reduction in bar thickness after surface preparation and finishing and polishing (FP) protocols. Based on these findings, specimens assigned to FP protocols were initially fabricated with an increased thickness (2 × 8 × 1.3 mm), to compensate for material removal, whereas control specimens (no FP) were prepared with final dimensions (2 × 8 × 1.0 mm). After completion of all FP procedures, all specimens were standardized to a final thickness of 1.0 mm, which was confirmed using a high-precision digital caliper (Digital Absolute AOS, Mitutoyo, Kanagawa, Japan). This approach ensured that the mini-flexural strength test was performed on specimens with identical final dimensions, thereby minimizing the influence of specimen geometry on flexural strength outcomes. Increased initial thickness in the FP groups was defined based on a pilot study to compensate for material removal during finishing and polishing procedures, following a methodology similar to that previously described [28].

The blocks were sectioned with a double-sided diamond disk (Microdont, São Paulo, Brazil; No. 34,570) mounted on a straight handpiece connected to a micromotor (LB100, Beltec, São Paulo, Brazil) under air/water irrigation (Fig. 1). In addition, 44 additional samples per group (4 × 4 × 4 mm) were prepared for optical profilometry (*n* = 10 per group) and scanning electron microscopy (*n* = 1 per group) analyses according to the factors “ceramic material” and “finishing and polishing” (ISO 4287:1997 + A1:2009). All bars were grounded and polished with water-abrasive sandpaper of increasing grit size (#800 and #1200, 3 M ESPE, Irvine, CA, USA). The final dimensions of all specimens were verified using a high-precision digital caliper (Digital Absolute AOS, Mitutoyo, Kanagawa, Japan). Subsequently, all specimens were cleaned in an ultrasonic bath (Cristófoli Equipamentos de Biossegurança LTDA, Paraná, Brazil) with distilled water for five minutes and then air dried. All specimen preparation procedures were conducted under controlled laboratory conditions (temperature: 23 ± 2 °C; relative humidity: 50 ± 10%) to minimize environmental variability. After FP protocols, all bars were the same thickness of 1 mm.

### Finishing / polishing protocol

The bars had their treatment surface marked with a permanent marker to ensure better visualization of the area subjected to FP and to promote uniform material removal. The bars were positioned in a heavy silicone matrix to ensure stabilization during all procedures. All finishing and polishing protocols were performed by a single calibrated operator. Prior to the experimental procedures, the operator underwent a calibration process involving repeated execution of all finishing and polishing protocols on pilot specimens until consistent material removal and surface patterns were achieved. The consistency of the procedure was verified by measuring specimen thickness after FP and ensuring deviations within ± 0.05 mm. A consistent light manual pressure was applied during all procedures, previously trained using a precision scale to standardize the applied force (~ 2 N), and a unidirectional motion was maintained along the entire length of the bar to ensure uniform wear. The polishing time for each step was strictly controlled using a digital stopwatch.

Surface finishing and polishing procedures were performed using a low-speed handpiece (approximately 10,000–15,000 rpm), following the manufacturers’ recommendations for each system, under continuous water cooling when applicable. At each polishing stage, the samples were rinsed with pressurized water for 1 min. The experimental groups were defined as follows:

- Control (C): No treatment.
- Diamond bur (B): Surface finishing was performed using cylindrical diamond burs (#2215-FG, 90–120 μm, KG Sorensen, Cotia, Brazil) with a high-speed handpiece under constant water cooling, following a standard unidirectional motion along the entire length of the bar for 10 s, until the samples reached a thickness of 1 mm. The burs were replaced with every 10 samples.
- Diamond bur + Polyurethane rubber tips impregnated with diamond particles (BD): The samples were abraded with the cylindrical diamond bur, as previously described, for 7 s. Then, polishing was performed using polyurethane rubber tips impregnated with diamond particles (Premium Compact, Dhpro, Paraná, Brazil) according to the manufacturer’s instructions (HZ1DL for wear, HZ2DL for pre-polishing, and HC3DL for high gloss), for 10 s each, until reaching a thickness of 1 mm.
- Diamond bur + Abrasive rubbers impregnated with silica (BS): The samples were abraded with the cylindrical diamond bur, as previously described, for 7 s. Then, polishing was performed using abrasive rubbers impregnated with silica (Jiffy Polisher, Ultradent, Brazil) according to the manufacturer’s instructions (coarse disc for wear, medium disc for pre-polishing, and fine disc for shine), for 10 s each, until reaching a thickness of 1 mm.

The final thickness of each bar was measured using a digital caliper (Eccofer, Curitiba/Paraná, Brazil) at three different points. Samples with a final thickness of less than 0.9 mm were excluded. Subsequently, all samples were cleaned in an ultrasonic bath with distilled water at 37 °C for 5 min to remove polishing residues.

### Aging

Half of the samples were stored in distilled water at 37 °C for 24 h (no aging) by a single operator blinded to the finishing and polishing protocols. Storage was carried out in an incubator with controlled temperature (37 ± 1 °C) to ensure environmental stability. The remaining samples were thermocycled for 10,000 cycles between 5 °C and 55 °C with 30 s immersion and 5 s transition (Nova Ethics, São Paulo, SP, Brazil) [29].

### Mini-flexural strength test

All specimens were subjected to a three-point mini flexural (σ) test using a universal testing machine (Odeme Dental Research ISO150, Luzerna, SC, Brazil). A single operator, blinded to the finishing and polishing protocols and aging conditions, performed the tests. All tests were performed in a temperature-controlled environment (23 ± 2 °C), and the testing machine was calibrated prior to the experiments according to the manufacturer’s recommendations.

To determine the thickness, each specimen was measured at three different points, and the arithmetic mean of these values was calculated to determine the final flexural strength per specimen. A metal fixture specifically designed for the dimensions of the bars was used for positioning. Each specimen was supported by two pins (2.0 mm in diameter) spaced 6 mm apart, with the treated surface facing down, while a load pin was placed in the center of the upper surface.

The test was performed at a displacement speed of 1.0 mm/min using a 100 kgf load cell. The flexural strength (MPa) was calculated based on the critical load at specimen failure using the following equation:

$$\mathrm{RF}=31\mathrm F/(2\mathrm{wh}^2)$$

where RF is the flexural strength (MPa), l is the distance (mm) between the lower pins, F is the critical load (N) at failure, w is the specimen width (mm), and h is the specimen thickness (mm).

### Optical profilometry

Surface roughness was assessed by a second examiner blinded to the FP protocols. The examiner was previously calibrated, and intra-examiner reliability was assessed by repeating measurements on 20% of the samples, yielding an intraclass correlation coefficient (ICC) > 0.90. A non-contact optical profilometer (CCI MP, Taylor Hobson, England) was used for analysis in conjunction with Talysurf CCI software (Taylor Hobson, Leicester, England).

Each sample was individually positioned on a glass plate coated with utility wax and placed under the reading lens of the profilometer. Measurements were taken using a 50× objective with a numerical aperture of 0.4, a scan speed of x1 in XYZ mode and a cut-off of 8 μm, covering an area of 0.34 × 0.34 mm². Each sample was scanned at three different points, and the arithmetic mean of these values was calculated to determine the final roughness value per sample.

Surface roughness was evaluated using the Sa parameter, which provides a three-dimensional areal characterization of surface texture and is considered more representative of complex surfaces obtained after finishing and polishing procedures. Unlike profile-based parameters such as Ra, Sa captures variations across the entire analyzed area [30].

### Scanning electron microscope (SEM)

The surfaces were first coated with gold particles for 15 s to obtain a layer thickness of 80 Å. Surface analysis was then performed using a scanning electron microscope (SEM) (TM3030, HITACHI, SP, Brazil) at 240x, 5000x, and 10,000x magnifications to evaluate the surface modifications induced by the different factors studied. SEM analysis was conducted for qualitative assessment only, and no statistical comparisons were performed. All images were acquired under standardized operating conditions (accelerating voltage, working distance, and magnification) to ensure consistency across samples. 

### Statistical analysis

A post hoc power analysis was performed based on the observed effect sizes derived from the ANOVA model for the primary outcome (flexural strength), considering an alpha level of 0.05. Statistical assumptions were assessed before statistical analysis. Data distribution was checked using the Shapiro-Wilk normality test. Normal distribution was observed for all groups tested (*p* > 0.05). Although the study design included three factors (material, finishing/polishing protocol, and aging), two-way ANOVA models were applied separately for each material, since the materials were not intended to be directly compared due to their distinct compositions and structural characteristics.

Mini-flexural strength data were analyzed by two-way analysis of variance (ANOVA) and Tukey’s test (α = 0.05), taking into account the factors “aging” and “finishing/polishing”. The Statistix program (Analytical Software Inc., version 8.0, 2003) was used for these analyses.

The chi-squared test, followed by the Bonferroni test, was used to calculate the p-value for the Weibull module. Statistical analysis was performed using Minitab software (version 17, 2013, Minitab, State College, PA). The significance level was set at 5%.

Weibull modulus and characteristic strength (mean and 95% CI) were determined using the following equation:

$$\\\mathrm{In}\left[\mathrm{In}\left(1/\left(1-\mathrm F\right)\right)\right]=\mathrm m\;\mathrm{In}\left(\mathrm\sigma\right)-\mathrm m\;\mathrm{In}\left(\sigma o\right)$$

Where F is the probability of failure, σ is the flexural strength, σ₀ is the characteristic strength (the strength at which the probability of failure is approximately 63%), and m is the Weibull modulus.

Optical profilometry was assessed by two-way analysis of variance (ANOVA) and Tukey’s test (α = 0.05), with the factors “finishing and polishing” and “ceramic material”. The Statistix program (Analytical Software Inc., version 8.0, 2003) was used for these analyses.

## Results

The results of the Shapiro-Wilk and Levene tests indicated normal and homogeneous data distribution (*p* > 0.05).

### Mini-flexural strength

**Fig. 2:**
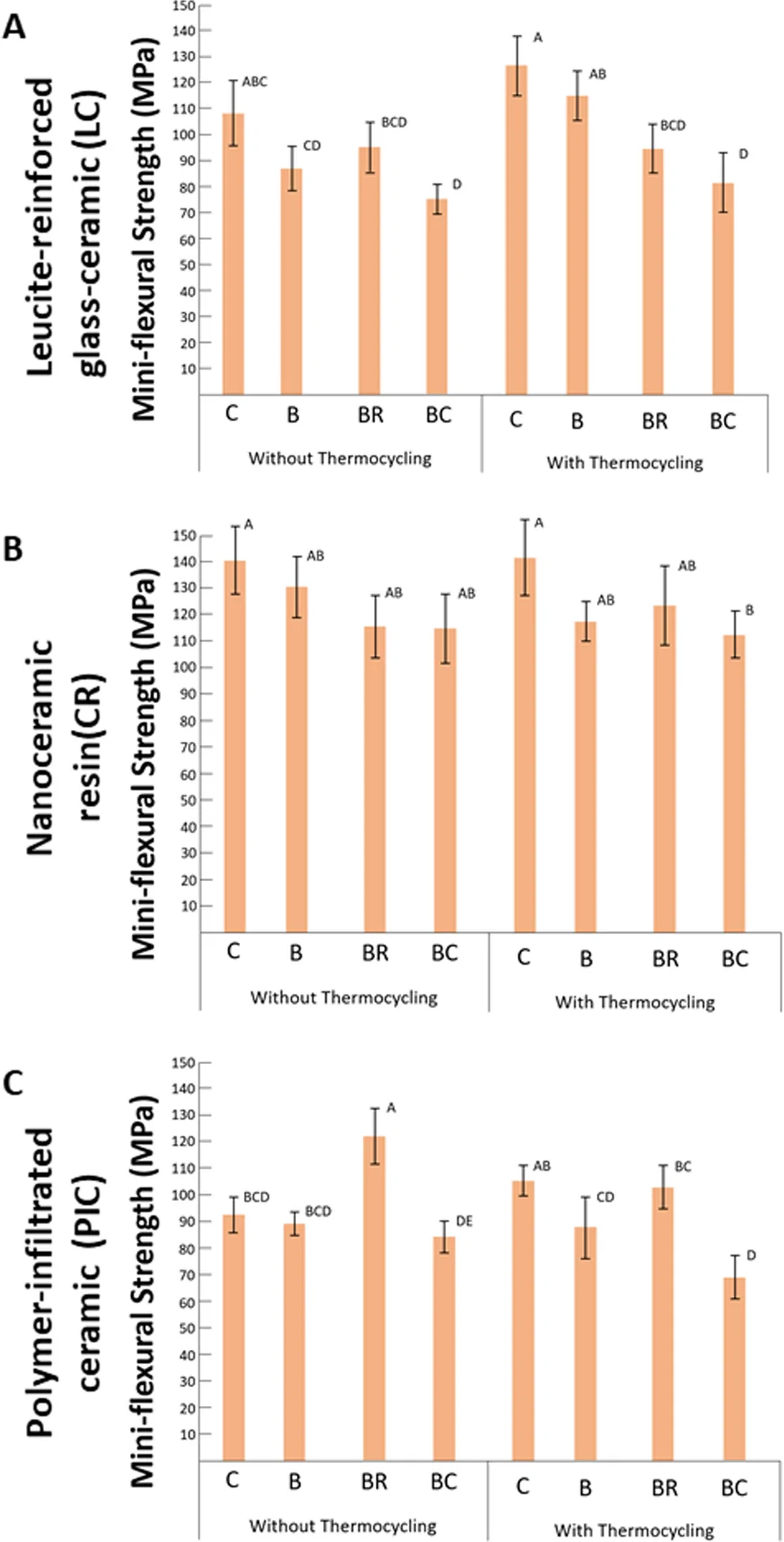
Means and standard deviations for the mini-flexural strength of Leucite-reinforced glass-ceramic group (**A**), Nanoceramic resin group (**B**), and Polymer-infiltrated ceramic group (**C**)

The effect size analysis revealed a large effect for finishing/polishing protocols (η^2^_p_ = 0.33), a moderate effect for aging (η^2^_p_ = 0.095), and a small-to-moderate effect for their interaction (η^2^_p_ = 0.07). Based on these values, the estimated statistical power exceeded 0.99 for finishing/polishing, ranged from 0.90 to 0.95 for aging, and was between 0.75 and 0.85 for the interaction effect. The means and standard deviations of the mini-flexural strength for the three materials are available in Fig. 2.

Two-way ANOVA revealed material-specific behaviors. For the CR, only the “finishing/polishing” factor was statistically significant (*p* = 0.0002). The control group, both aged (141.36 MPa^A^) and unaged (140.51 MPa^A^), presented the highest flexural strength values, statistically similar to the other groups, except for the aged BD group (112.00 MPa^B^), which presented significantly lower values.Aging and interaction effects were not significant for CR.

For the PIC, both main factors (“finishing/polishing” and “aging”) and their interaction were statistically significant (*p* < 0.05). The non-aged BS group demonstrated the highest strength (121.79 MPa^A^), while the aged BD group showed the lowest (68.72 MPa^E^). Overall, aging negatively impacted the mechanical strength of PIC.

For the LC, both “finishing/polishing” (*p* = 0.0000) and “aging” (*p* = 0.0000) were significant, but their interaction was not (*p* = 0.418). Interestingly, aging increased the mechanical strength of LC. The aged control group (125.66 MPa^A^) presented the highest flexural strength values, statistically similar to the aged B group (114.75 MPa^AB^) and the non-aged control group (108.11 MPa^ABC^). These, in turn, were similar to both the non-aged BS group (95.12 MPa^ABCD^) and the aged BS group (94.48 MPa^ABCD^). The lowest flexural strength values were found in the BD groups, both aged (81.34 MPa^D^) and non-aged (74.99 MPa^D^), followed by the non-aged B group (86.69 MPa^CD^).

**Fig. 3:**
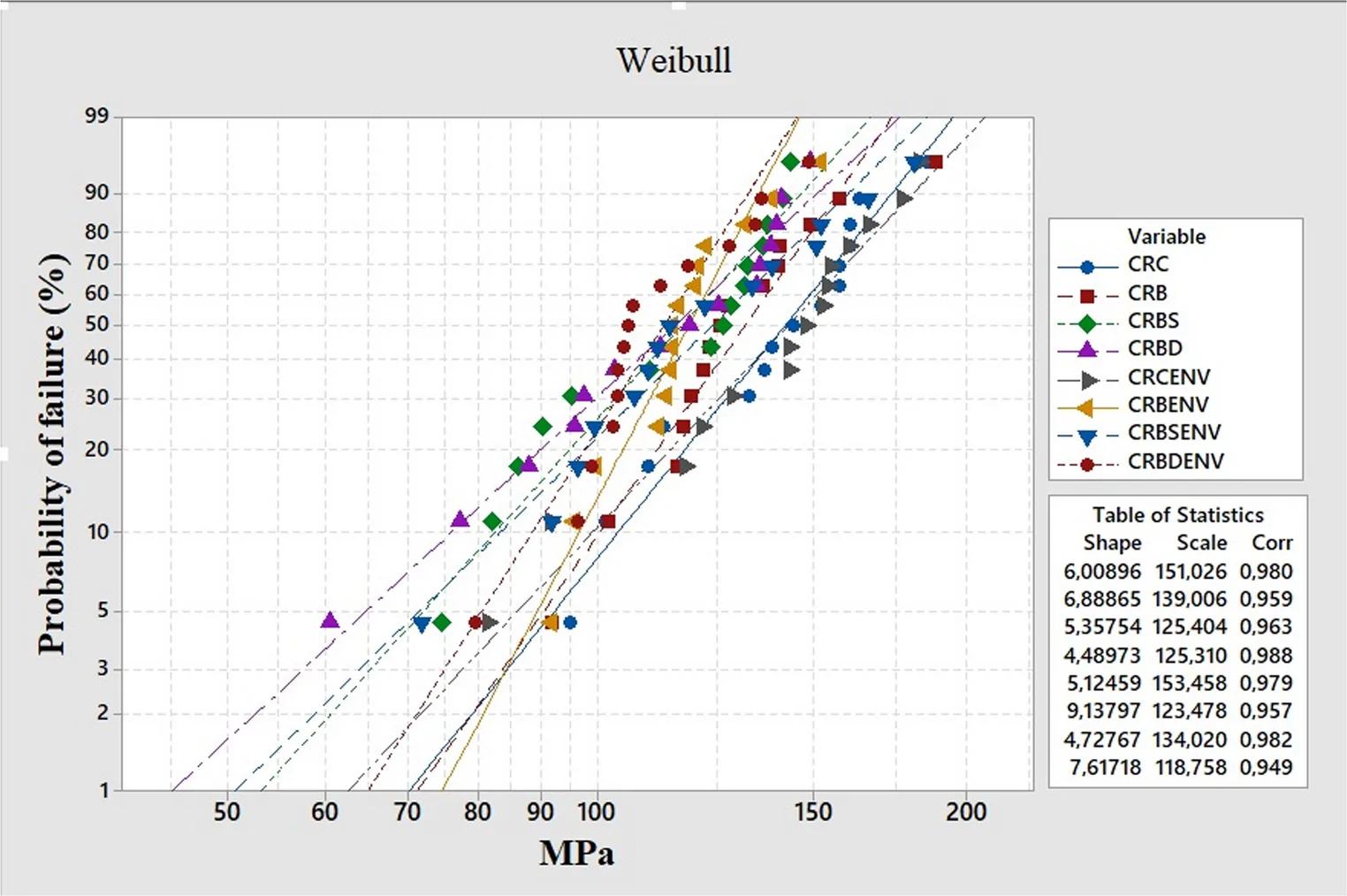
Weibull plots of flexural strength for Nanoceramic resin

**Fig. 4:**
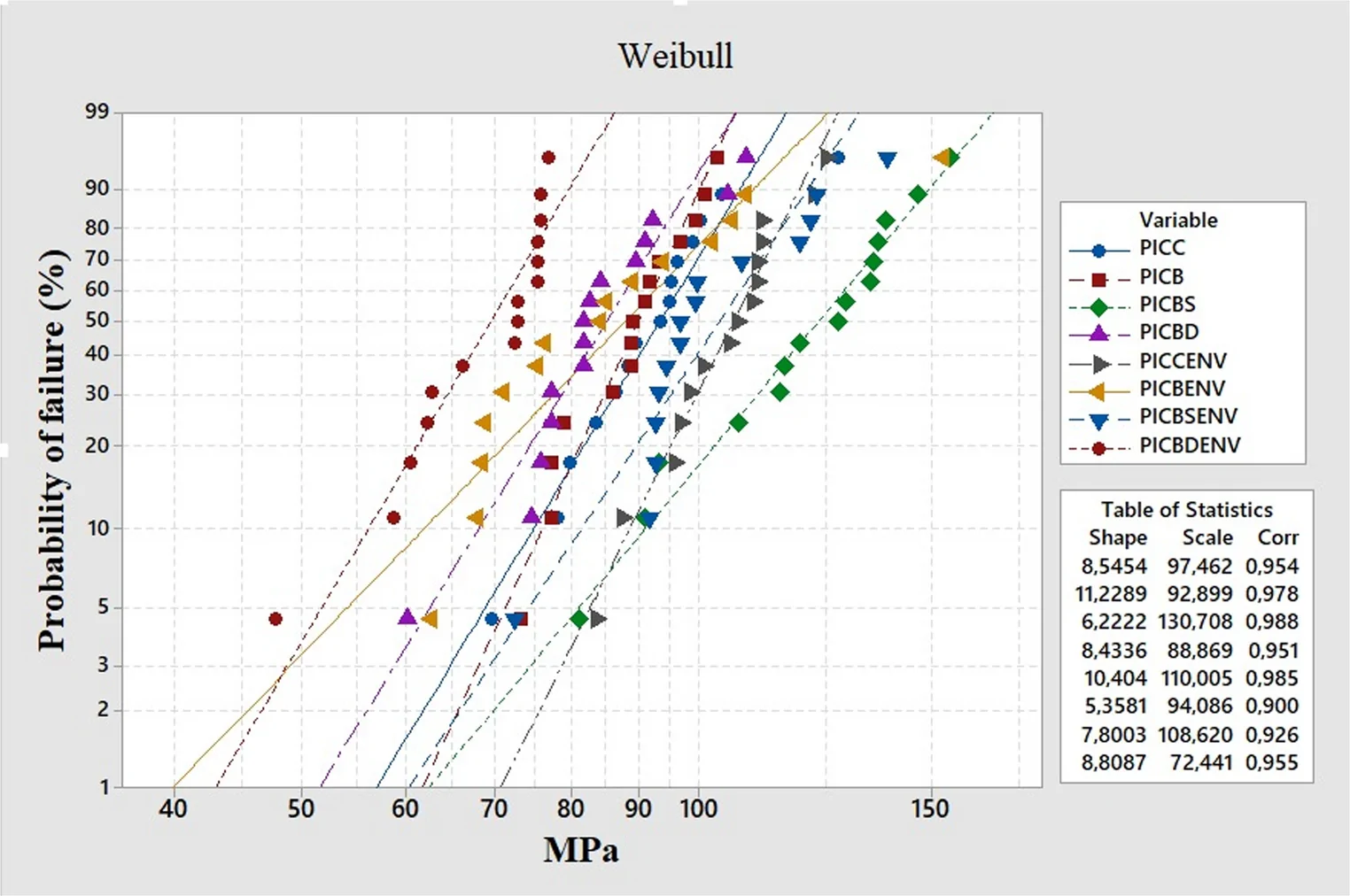
Weibull plots of flexural strength for Polymer-infiltrated ceramic

**Fig. 5:**
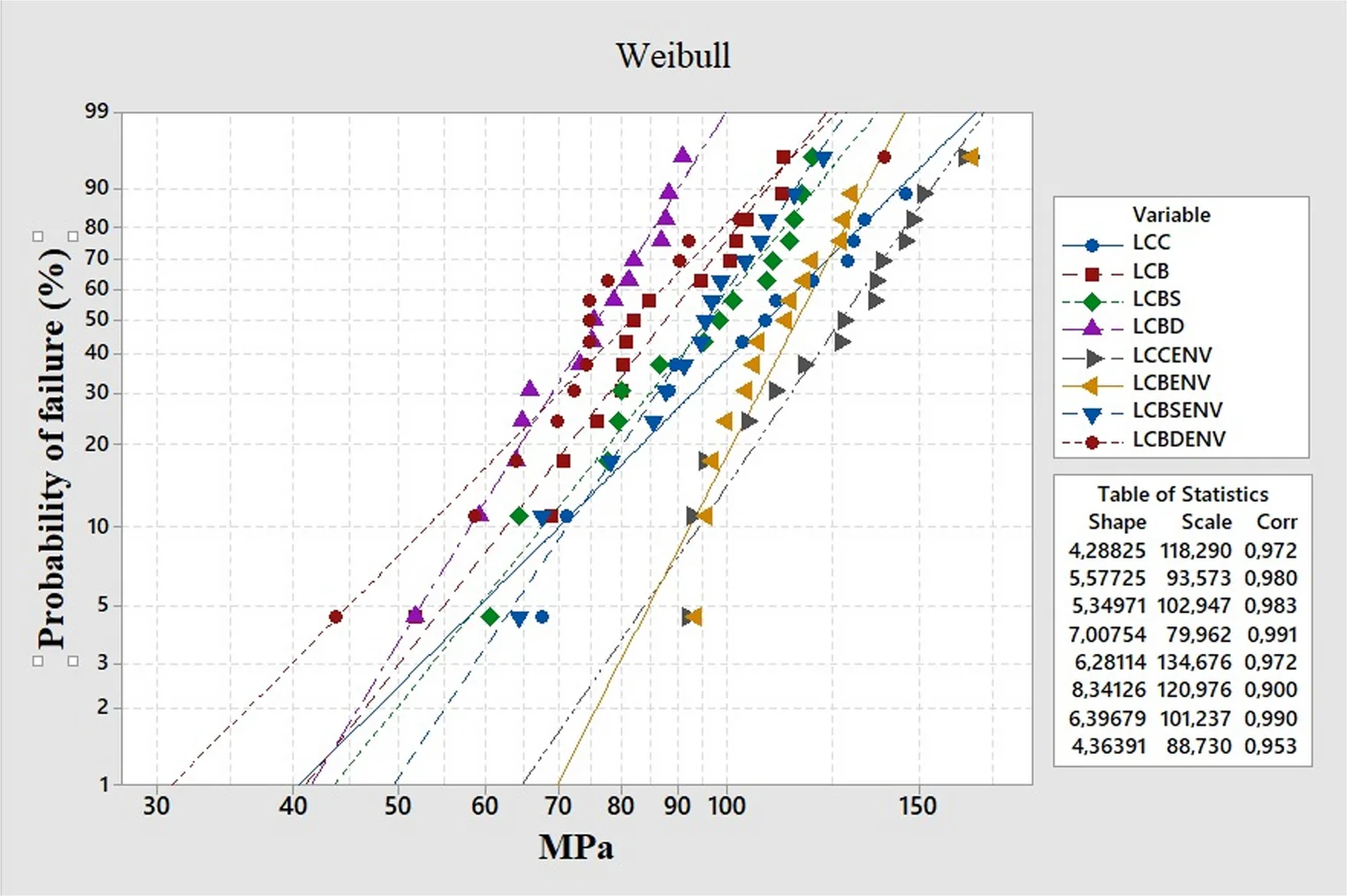
Weibull plots of flexural strength for Leucite-reinforced glass-ceramic

Regarding the Weibull analysis, the modulus (m) was statistically similar across groups for CR (*p* = 0.101) and PIC (*p* = 0.009) but differed significantly for LC (*p* = 0.007), with the aged B group presenting the highest value. The trends for characteristic strength (σ₀) closely followed the mini-flexural strength outcomes across all materials (Table 2; Figs. 3, 4 and 5).

**Table 2:** Mean mini-flexural strength (MPa) with standard deviations, Weibull Modulus (m), Weibull characteristic strength, and confidence intervals for all experimental groups (*n* = 15)

Groups			Flexural strength (MPa)	Weibull modulus (m)	95% CI for (m)	Characteristic resistance (σo)	95% CI for (σ_o_) (MPa)
Resin	Finishing / polishing	Aging					
LC	C	Without	108.11^ABC^ ± 29.54	4.28	2.97 ± 6.17	118.29^AC^	104.35 ± 134.08
	B		86.69^CD^ ± 17.27	5.57	3.60 ± 8.62	93.57^CD^	85.04 ± 102.96
	BS		95.12^BCD^ ± 19.41	5.34	3.20 ± 8.92	102.94^BC^	93.20 ± 113.70
	BD		74.99^D^ ± 11.79	7.00	4.26 ± 11.52	79.96^DE^	74.11 ± 86.27
	C	With (_TC_)	125.66^A^ ± 22.88	6.28	4.10 ± 9.60	134.67^A^	123.71 ± 146.61
	B		114.75^AB^ ± 19.00	8.34	6.85 ± 10.15	120.97^AB^	113.01 ± 129.49
	BS		94.48^BCD^ ± 19.28	6.39	4.08 ± 10.01	101.23^BC^	93.14 ± 110.03
	BD		81.34^D^ ± 23.24	4.36	3.28 ± 5.79	88.72^CE^	78.30 ± 100.54
CR	C	Without	140.51^A^ ± 25.92	6.00	3.78 ± 9.55	151.02^A^	138.22 ± 165.01
	B		130.44^AB^ ± 23.35	6.88	5.27 ± 8.99	139.00^AB^	128.39 ± 150.49
	BS		115.98^AB^ ± 23.92	5.35	3.11 ± 9.28	125.40^AD^	113.57 ± 138.46
	BD		114.47^AB^ ±26.57	4.48	2.55 ± 7.87	125.31^AE^	111.27 ± 141.11
	C	With (_TC_)	141.36^A^ ± 28.85	5.12	2.89 ± 9.07	153.45^A^	138.26 ± 170.35
	B		117.38^AB^ ± 15.54	9.13	6.80 ± 12.27	123.47^BCDE^	116.34 ± 131.04
	BS		123.06^AB^ ± 30.03	4.72	3.26 ± 6.84	134.02^AC^	119.63 ± 150.14
	BD		112.00^B^ ± 17.97	7.61	5.57 ± 10.41	118.75^BCDE^	110.57 ± 127.54
PIC	C	Without	92.40^BCD^ ± 13.43	8.54	6.71 ± 10.87	97.46^BC^	91.38 ± 103.94
	B		89.01^BCD^ ± 9.16	11.22	7.40 ± 17.03	92.89^CD^	88.58 ± 97.42
	BS		121.79^A^ ± 21.35	6.22	3.76 ± 10.26	130.70^A^	119.98 ± 142.38
	BD		84.19^DE^ ± 12.04	8.43	6.20 ± 11.46	88.86^CD^	83.33 ± 94.77
	C	With (_TC_)	105.08^AB^ ± 11.55	10.40	6.99 ± 15.48	110.00^B^	104.49 ± 115.80
	B		87.58^CD^ ± 23.42	5.35	4.35 ± 6.59	94.08^BD^	84.67 ± 104.54
	BS		102.61^BC^ ± 16.59	7.80	5.86 ± 10.37	108.62^AB^	101.25 ± 116.52
	BD		68.72^E^ ± 16.89	8.80	4.60 ± 16.84	72.44^E^	68.17 ± 76.97

Different capital letters superscript indicates significant difference between groups for fracture resistance. Different lowercase letters superscript indicates a significant difference between groups for the characteristic resistance

### Surface roughness

The power, calculated using the OpenEpi website with a 95% confidence interval for the comparison of two means, was 100%. The Shapiro–Wilk and Levene tests were performed for each group and confirmed that the data followed a normal distribution and showed homogeneity, respectively (*p* < 0.05).

**Fig. 6:**
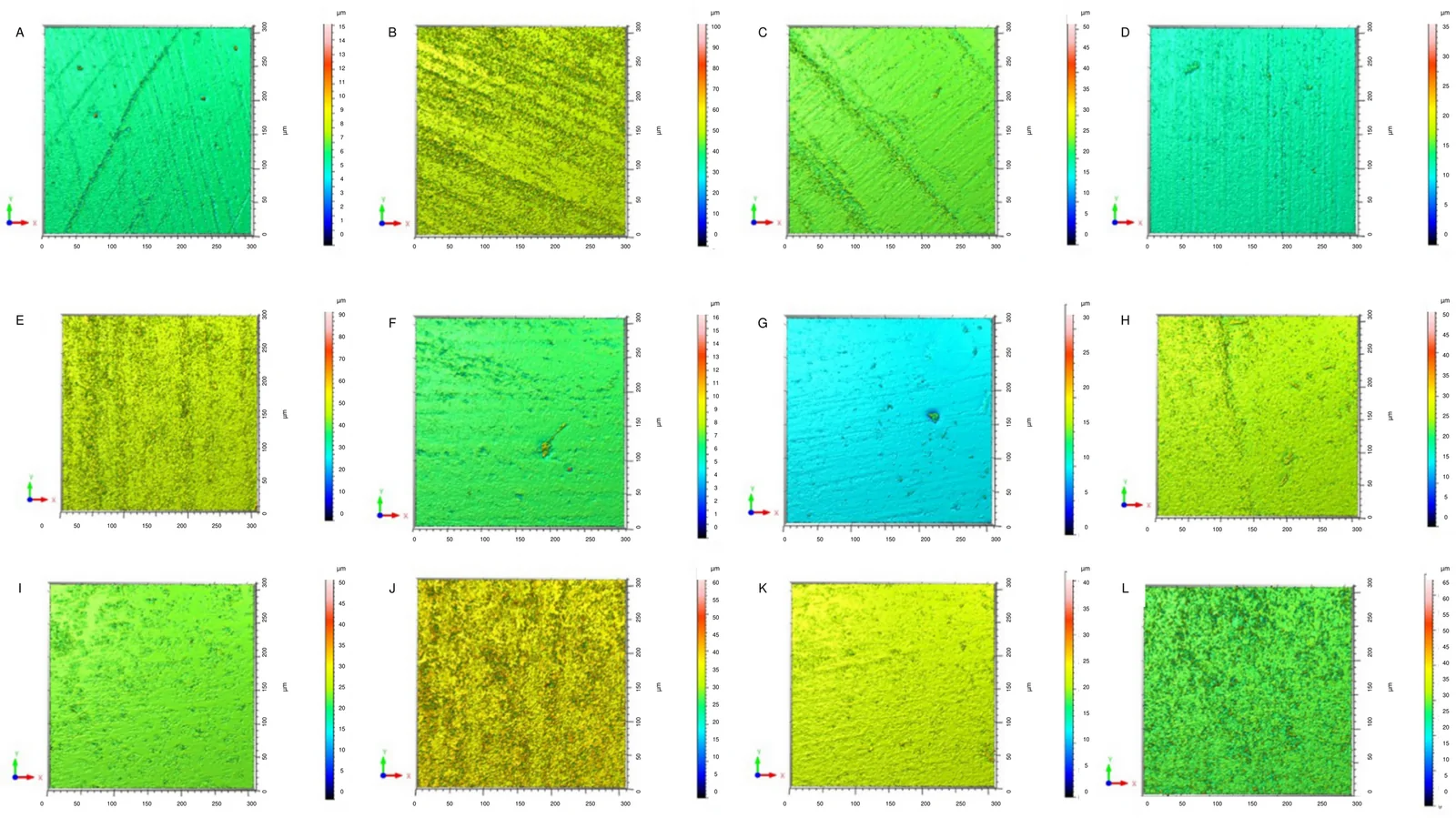
SEM (250×) of the surface of resin materials with different finishing/polishing protocols. **A**: CR_C group; **B**: CR_B group; **C**: CR_BS group; **D**: CR_BD group; **E**: PIC_C group; **F**: PIC_B group; **G**: PIC_BS group; **H**: PIC_BD group; **I**: LC_C group; **J**: LC_B group; **K**: LC_BS group; **L**: LC_BD group. The use of diamond burrs (**B**, **F**, **J**) induced surface irregularities compared to the control groups (**A**, **E**, **I**). Groups treated with diamond burrs followed by composite resin rubber polishers (**C**, **G**, **K**) had improved surface smoothness compared to the exclusive use of diamond burrs, while groups treated with diamond burrs and ceramic rubber polishers (**D**, **H**, **L**) had the smoothest surfaces, which follows the surface roughness results. Differences in porosities may be due to material composition, imaging angle, or surface treatment

The 2-way ANOVA revealed that both the factors “finishing and polishing” and “ceramic material” were statistically significant (*p* = 0.0001) for the Sa (surface area roughness. In addition, the interaction between these two factors was statistically significant (*p* = 0.0001), indicating that the effect of the finishing/polishing protocol on roughness depended on the type of ceramic material used (Table 3; Fig. 6).

**Table 3:** Means and standard-deviations of roughness (Sa, µm) for all groups, as well as statistical analysis (*)

Ceramic material	Finishing/polishing	Sa Mean ± SD
LC	C	0.3796^C^ ± 0.1438
	B	2.6110^AB^ ± 1.5991
	BD	0.6701^C^ ± 0.2401
	BS	2.8116^AB^ ± 1.5991
CR	C	0.1012^C^ ± 0.0338
	B	3.4475^A^ ± 0.5697
	BD	0.3204^C^ ± 0.1297
	BS	0.3655^C^ ± 0.1904
PIC	C	0.3125^C^ ± 0.2830
	B	2.2498^B^ ± 0.7970
	BD	0.1489^C^ ± 0.0657
	BS	0.5560^C^ ± 0.1695

(*) Different capital letters as superscripts indicate statistically significant differences among groups. For the ceramic materials, group LC showed the highest roughness values, while groups CR and PIC were statistically similar. Among the finishing/polishing protocols, the B group exhibited the highest roughness, followed by BS, whereas C and BD produced the lowest values. In the interaction analysis, the CR_B group presented the highest roughness, which was statistically similar to LC_BS and significantly greater than PIC_B and LC_B, while the CR_C group recorded the lowest values, comparable to PIC_BD, PIC_C, CR_BD, CR_BS, LC_C, PIC_BS, and LC_BD

**Fig. 7:**
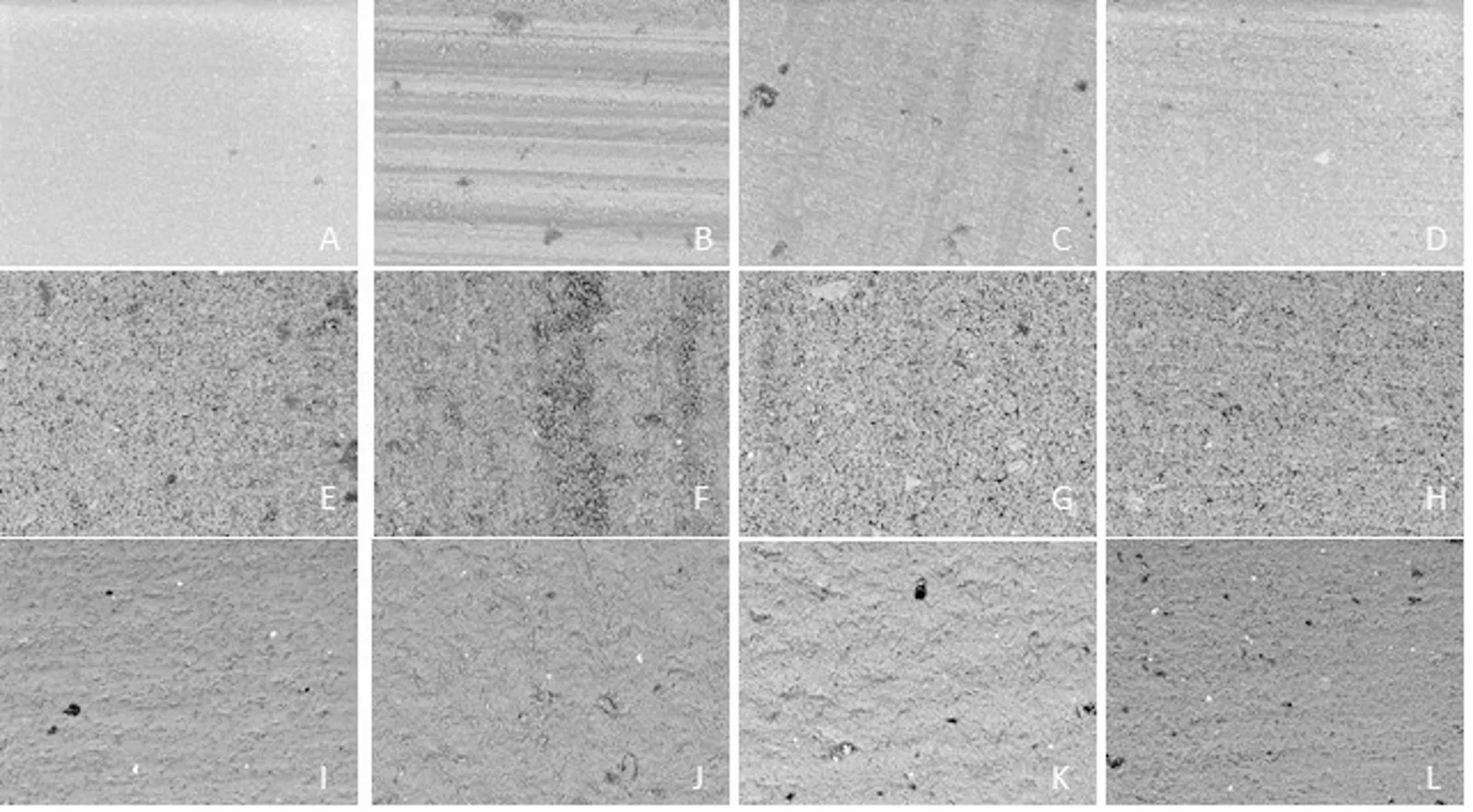
Representative profilometry images of the surface topography of the tested specimens. **A**–**D**: Nanoceramic resin (CR)—**A**, control (C); **B**, diamond burr (B); **C**, diamond bur + polyurethane rubber tips impregnated with diamond particles (BD); **D**, diamond bur + abrasive rubbers impregnated with silica (BS). **E**–**H**: Polymer-infiltrated ceramic (PIC)—E_C; F_B; G_BD; H_BS. **I**–**L**: Leucite-reinforced glass-ceramic (LC)—I_C; J_B; K_BD; L_BS. Among the ceramics, group LC (I–L) presented the highest roughness values, while CR (A–D) and PIC (E–H) were statistically similar. Regarding the finishing/polishing protocols, group B (B, F, J) produced the highest roughness values, followed by BS (D, H, L). Groups C (A, E, I) and BD (C, G, K) resulted in the smoothest surfaces. The CR_B combination (B) exhibited the highest overall roughness, whereas CR_C (A) demonstrated the lowest roughness values

Regarding the ceramic material, group LC exhibited the highest mean roughness values (Sa = 1.6181^A^), while groups CR and PIC did not differ statistically from each other. For the finishing/polishing protocols, the B group produced the highest roughness values (Sa = 2.7694^A^), followed by the BS group (Sa = 1.2444^B^), whereas the C and BD groups showed the lowest roughness values.

Analysis of the interaction between material and treatment indicated that the CR_B combination yielded the highest roughness values (Sa = 3.4475^A^), which were statistically similar to those observed in the LC_BS group (Sa = 2.8116^AB^). These were followed by the PIC_B (Sa = 2.2498^B^) and LC_B (Sa = 2.6110^AB^) groups. In contrast, the CR_C group presented the lowest roughness averages (Sa = 0.1012^C^), which were statistically similar to the PIC_BD, PIC_C, CR_BD, CR_BS, LC_C, PIC_BS, and LC_BD groups.

### Scanning electron microscope

The groups subjected to surface treatment protocols exhibited alterations in surface topography compared to the control group (Fig. 7). Greater surface uniformity was observed in the groups that underwent a finishing procedure using diamond burs followed by polishing with diamond polishers (Fig. 7-D, H and L). In contrast, the groups subjected only to finishing with diamond burs (Figs. 7-F and J and 7-B), and the groups subjected to diamond burs followed by abrasive polishers (Fig. 7-C, G and K) showed pronounced surface irregularities.

## Discussion

The present study evaluated the impact of different finishing and polishing protocols, combined with aging, on the flexural strength and surface roughness of CAD/CAM restorative materials. Due to the dimensional limitations of commercial CAD/CAM blocks, which often prevent the fabrication of standardized specimens (2 × 2 × 25 mm) according to ISO 4049, the mini-flexural strength test was employed, following the methodology described by Vila-Nova et al. (2020) [31] and Carvalho et al. (2022) [32].

The first hypothesis—that finishing/polishing protocols do not affect the mini-flexural strength of CAD/CAM restorative materials—was rejected. Significant variations in strength were observed depending on the surface treatment applied. The materials selected for this study differ in their structural composition: LC is a leucite-reinforced glass-ceramic (1–5 μm crystals at ~ 45 vol%) [33]; CR is a resin nanoceramic composite composed of silica and zirconia nanoclusters, without a glassy phase available for acid etching; and PIC is a polymer-infiltrated ceramic network, with approximately 86 wt% leucite ceramic [34].

The influence of finishing and polishing protocols was more pronounced in PIC. The use of silica-impregnated abrasive rubbers resulted in higher flexural strength values. Given the heterogeneous structure of PIC materials, characterized by the coexistence of interpenetrating ceramic and polymer networks, surface defects generated during finishing and polishing procedures may alter local stress distribution and compromise stress transfer between both phases [35, 36]. These defects can act as preferential sites for stress concentration, facilitating crack propagation [35, 36]. Consistent with the present findings, Alhassan et al. (2023) reported higher flexural strength values for Vita Enamic after polishing procedures, highlighting the influence of surface integrity on the mechanical behavior of PIC materials [35]. Therefore, the use of silica-impregnated abrasive rubbers may have better preserved the integrity of the PIC network by minimizing surface damage and limiting the formation of critical defects, which could explain the higher flexural strength values observed in the present study.

In contrast, for LC, the use of diamond-impregnated polishing systems resulted in lower flexural strength, despite producing smoother surfaces. Although lower surface roughness is generally associated with improved mechanical performance, the relationship between surface quality and strength is highly material-dependent. According to the literature, in leucite-reinforced glass-ceramics, mechanical behavior is primarily influenced by the size and distribution of critical flaws rather than by surface topography alone [37]. Therefore, a smoother surface may not necessarily translate into higher strength. Previous studies have suggested that diamond abrasives may induce subsurface damage during finishing procedures, generating microdefects which may not be necessarily reflected by conventional roughness parameters [38]. These defects may act as stress concentrators and fracture origins under flexural loading, leading to premature failure [16]. Thus, the reduction in flexural strength observed for LC may be associated with subsurface damage introduced during polishing rather than with the resulting surface roughness itself. However, fractographic analyses were not performed in the present study, and this hypothesis should be interpreted with caution.

In addition, another possible explanation is that the damage introduced by the initial diamond bur was not completely removed by the subsequent polishing procedures. In brittle glass-ceramics, grinding may generate a subsurface damaged layer containing microcracks that extend beyond the surface irregularities detected by roughness measurements. Although polishing procedures can effectively reduce surface roughness, they may be insufficient to eliminate these deeper defects. Consequently, residual subsurface flaws may continue to act as fracture origins under flexural loading, explaining the lower flexural strength observed despite the smoother surfaces.

For CR, the tested protocols did not result in statistically significant changes in mechanical strength, indicating higher stability. Unlike brittle glass-ceramics, such as LC, resin-based CAD/CAM materials exhibit higher tolerance to subcritcial crack growth because the polymeric phase can absorb and redistribute stresses, which could potentially reduce crack propagation, limiting the detrimental effect of surface defects on mechanical performance [39]. Consequently, defects potentially introduced during finishing procedures may have a less pronounced influence on flexural strength, which could explain the similar mechanical behavior observed among the tested polishing protocols. In addition, the high filler content and industrial post-curing process are known to contribute to the mechanical stability and wear resistance of these materials [39]. These findings reinforce that the effect of finishing and polishing protocols is strongly material-dependent, highlighting the importance of considering material composition when selecting clinical polishing strategies.

The second hypothesis—that aging does not influence the mechanical resistance of CAD/CAM materials—was also rejected. Aging significantly affected the flexural strength of the tested materials, with distinct responses depending on material composition. For PIC, a reduction in flexural strength was observed after thermocycling. This behavior may be associated with hydrolytic degradation processes involving both the polymer network and the ceramic–polymer interface, as previously reported for resin-containing CAD/CAM materials [3, 40]. Water uptake may promote plasticization of the polymer phase and gradual degradation of interfacial bonds, which could facilitate the formation of microdefects and compromise stress transfer between both phases [23, 24, 41]. As a result, thermocycling may facilitate the formation and propagation of microdefects at the ceramic–polymer interface, compromising stress transfer between both phases and ultimately reducing the mechanical strength of the material.

For LC, an increase in flexural strength was observed in some experimental conditions after aging. Although most studies report neutral or negative effects of aging on ceramic materials, variations in experimental conditions may occasionally lead to different outcomes. One hypothesis is related to the relaxation or redistribution of residual stresses introduced during manufacturing and machining procedures. Previous studies have shown that stress relaxation can improve the mechanical performance of dental glass-ceramics by reducing the influence of localized tensile stresses associated with pre-existing defects [42]. Therefore, it is possible that thermal aging contributed to a more favorable stress distribution within the material, resulting in the higher flexural strength values observed [43]. However, the underlying mechanisms—such as stress relaxation or redistribution—were not directly investigated in the present study.

A second hypothesis is related to the distribution of critical defects among the samples. Given the predominant inorganic structure of glass-ceramic materials, they are less susceptible to the hydrolytic damage observed in resin-containing CAD/CAM materials. Consequently, thermocycling may not necessarily result in a reduction in mechanical strength. Since the mechanical behavior of glass-ceramics is largely determined by the size and distribution of critical flaws, the increase in flexural strength observed in the present study may reflect differences in flaw distribution among the experimental groups rather than a direct strengthening effect of the aging procedure, although all specimens were randomly allocated [37]. Therefore, these explanations should be interpreted with caution and considered as hypotheses supported by existing literature rather than definitive conclusions. In contrast, CR showed no significant changes after aging, suggesting greater structural stability under the tested conditions. This behavior has been associated with the material’s microstructural characteristics, including high filler content and homogeneous distribution of nanofillers, which may contribute to reduced susceptibility to hydrolytic degradation [21, 44].

The third hypothesis—that finishing/polishing protocols do not affect surface roughness—was also rejected. Both the type of material and the polishing protocol significantly influenced surface roughness values, and a significant interaction between these factors was observed. Overall, LC exhibited higher roughness values compared to CR and PIC. Among the polishing protocols, the use of diamond burs alone resulted in the highest roughness, whereas diamond-impregnated polishing systems produced smoother surfaces. These findings are consistent with previous studies reporting that multi-step polishing systems are more effective in reducing surface irregularities [14, 25].

Although surface roughness is often associated with mechanical performance, the present results suggest that this relationship is not straightforward. Surface roughness parameters mainly describe the topographic irregularities of the external surface, whereas flexural strength is more directly governed by the population of structural flaws, including their size, depth, orientation, location, and distribution within the material. Therefore, smoother surfaces may not necessarily result in higher mechanical strength if finishing or polishing procedures induce or fail to remove critical subsurface defects [14–16]. Conversely, surfaces with slightly higher roughness may still exhibit favorable mechanical behavior when the flaw population remains less critical. This distinction may explain why some groups with lower roughness values did not show higher flexural strength, reinforcing that surface smoothness does not necessarily correlate with mechanical reliability [16, 20]. However, subsurface defects and flaw populations were not directly assessed in the present study and should be interpreted as potential contributing mechanisms rather than confirmed causes.

SEM analysis supported the surface roughness findings by illustrating differences in surface morphology among the groups. The polished groups exhibited more uniform surfaces, while those subjected only to finishing procedures showed more pronounced irregularities. SEM analysis was used as a qualitative tool to illustrate surface morphology and to complement the quantitative roughness data obtained by optical profilometry. As only one representative specimen per group was analyzed, no statistical inference was intended from SEM observations. This approach is consistent with previous studies in dental materials research, where SEM is used as a descriptive method to support quantitative findings [14, 25]. Therefore, these images should be interpreted as illustrative rather than definitive evidence of surface behavior.

The results of the present study highlight that the relationship between surface treatment, aging, and mechanical performance is complex and dependent on material composition. These findings have important clinical implications, suggesting that finishing and polishing protocols should be selected according to the specific characteristics of each material rather than applied uniformly.

This study has limitations inherent to its in vitro design, as it does not fully replicate the complex oral environment, including thermal fluctuations, pH variation, and cyclic loading. Although specimen thickness was standardized, differences in material removal during finishing and polishing could have influenced the distribution of surface and subsurface defects, potentially affecting flexural strength and Weibull behavior. Therefore, clinical extrapolation and generalization to other materials should be approached with caution.

Additionally, mechanistic interpretations related to hydrolytic degradation and stress redistribution were based on existing literature [16, 40] and were not directly evaluated in this study. The absence of microstructural analyses limits the ability to correlate surface alterations with mechanical performance. Further studies, including microstructural investigations and clinical trials, are required to validate these findings.

## Conclusion

Within the limitations of this in vitro study, finishing and polishing protocols significantly influenced the flexural strength and surface roughness of CAD/CAM materials, with material-dependent effects. Polyurethane rubber tips impregnated with diamond particles may compromise the mechanical strength of leucite-based ceramics, despite producing smoother surfaces. In contrast, silica-impregnated abrasive rubbers provided a favorable balance between surface smoothness and flexural strength for Polymer-infiltrated ceramic. Nanoceramic resin was not significantly affected by the polishing protocols. Aging showed material-specific effects, enhancing the flexural strength of leucite-based ceramics while reducing that of Polymer-infiltrated ceramic. Furthermore, achieving lower surface roughness does not necessarily translate into improved flexural strength. 

## Data Availability

No datasets were generated or analysed during the current study.
